# Endometriosis of the vesico-vaginal septum: a rare and unusual localization (case report)

**DOI:** 10.1186/s12905-020-01047-w

**Published:** 2020-08-14

**Authors:** Yassir Ait Benkaddour, Affaf El Farji, Abderraouf Soummani

**Affiliations:** Department of obstetrics and gynecology, Mohammed VI University Hospital Center. Cadi Ayyad University, Marrakesh, Morocco

**Keywords:** Endometriosis, Deep pelvic endometriosis, Vesico-vaginal septum, Anterior deep endometriosis

## Abstract

**Background:**

We report a rare and unusual case of endometriosis in the vesico-vaginal septum. The location of this disease at this site is so uncommon that the literature about is very rare.

**Case presentation:**

A 41-year-old female was presented with urinary symptoms. There was history of caesarean section. Physical examination revealed an anterior vaginal wall mass. Pelvic MRI showed an inter vesico-vaginal mass, suggesting a leiomyoma. Surgical excision was performed by the vaginal route. There were no postoperative complications. Histopathology examination showed focal endometriosis.

**Conclusion:**

Endometriosis of the anterior compartment remains relatively rare; its localization to the vesico-vaginal septum (VVS) is very rare. With the occurrence of nonspecific cyclic urinary signs in women during periods of genital activity, endometriosis should be mentioned, especially in the presence of an antecedent of pelvic surgery.

## Background

Endometriosis is defined by the presence of endometrial glands and stroma outside the uterus. The estimated prevalence of this disease is unknown, but varies from 2 to 10% of women of reproductive age, to 50% of infertile women. Three types of endometriosis have been described, often associated with each other: peritoneal superficial endometriosis; ovarian endometrioma and; deep infiltrating endometriosis (DIE). The latter is defined as endometriosis that penetrates more than 5 mm under the peritoneal surface. Ovaries, ovarian fossa, uterosacral ligaments, Douglas’ Pouch, and rectovaginal septum are the most frequent localizations for endometriosis. DIE is the most severe type of the disease. We report a rare and unusual case of endometriosis in the vesico-vaginal septum. The rarity of this localization is attested in the literature with a few case reports.

## Case presentation

**Fig. 1:**
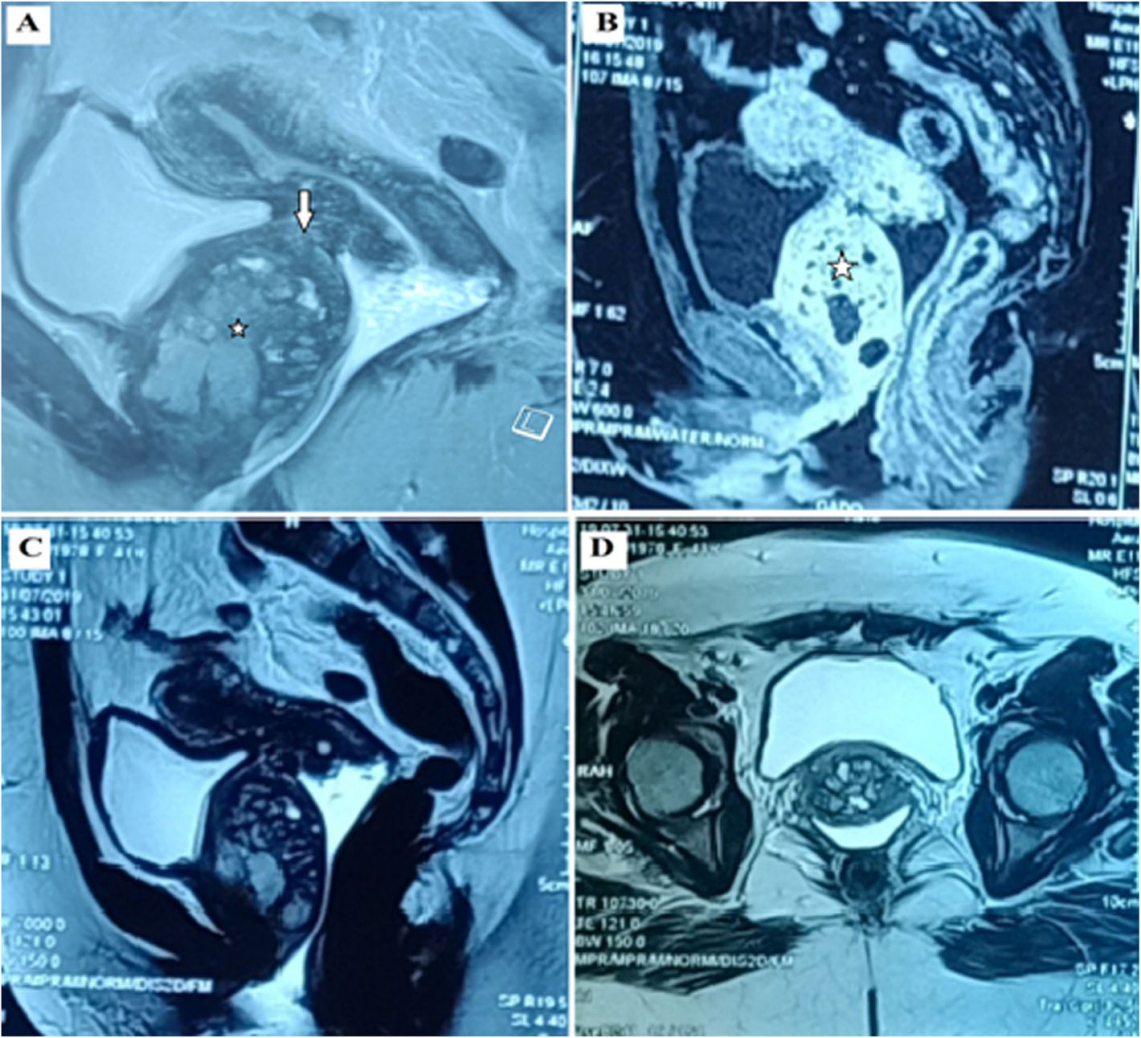
Pelvic MRI shows a pedicled inter vesico vaginal mass (60/48/38 mm) (star). This mass is connected to the anterior cervical stroma by a short pedicle measuring 5 mm in sagittal diameter and 6 mm in width (arrow), compressing the urethra and vagina and reaching the vaginal range. **a** Sagittal section in T1, the mass is isointense. **b** Sagittal section after injection, the mass takes the contrast in an intense and early manner. **c** T2 sagittal slice of heterogeneous signal with several cystic zones. **d** axial section in T2

**Fig. 2:**
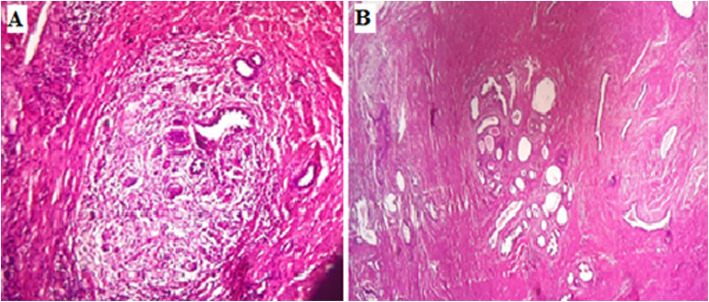
(**a**-**b**) At the histopathology examination, presence of a fibrous and muscular tissue, enclosing endometrial glands surrounded by cytogenic chorion

A 41-year-old woman, 4 gravida, 2 para, 2 Abortions with a history of C-section delivery. The patient presented with dysuria, pollakiuria, pelvic heaviness and an episode of acute urinary retention. Physical examination revealed a solid, well limited and fixed mass on the anterior vaginal wall. Pelvic ultrasound revealed a heterogeneous inter vesico-vaginal mass measuring 60/40 mm. MRI (Fig. 1) showed a pedicled inter vesico-vaginal mass (60/48/38 mm), isointense on T1, a heterogeneous signal on T2 with several cystic zones taking contrast in an early and intense rate suggesting cervical leiomyoma. The Surgical evaluation was recommended by the vaginal route due to the location of the mass. A *midline incision was carried* in the anterior *vaginal* wall*, 2 cm* below the *urethral meatus*. Mass resection was performed in two fragments, after adhesiolysis, which was difficult because of adhesion to the bladder, urethra and vaginal wall. A bladder breach was identified after resection of the mass and was immediately sutured. The removed tissue was a round, firm, 80/50/40 mm, gray tumor. Histopathology examination of the specimen showed that it was a focal endometriosis (Fig. 2**).** The urinary catheter was removed after 1 week. There were no postoperative complications.

## Discussion and conclusion

Endometriosis is a common, benign, chronic gynecological disorder, affecting 2–10% of women during periods of genital activity. Posterior involvement accounts for 90% of cases of deep endometriosis. The uterosacral ligament is the most frequent localization of deep infiltrating endometriosis (52.7%) [1, 2]. Anterior deep endometriosis is markedly less frequent than posterior involvement and essentially affects the vesicouterine pouch and bladder. These are the most frequent in case of adenomyosis, and in cases of previous caesarean section. They are associated in three-quarters of cases, with posterior involvement. Endometriosis of the vesicouterine pouch is the most common site of anterior DIE whereas VVS involvement is very rare [3, 4]. The development of endometriosis is not compatible with VVS, which is much more caudal. Anatomically, the VV septum does not extend beyond the cervix [4–6]. Three different etiologic hypotheses have been proposed [6, 7]: retrograde menstruation theory; extension of the adenomyosis from the anterior uterine wall, and metaplasia of subperitoneal mullerian remains located in the vesico-vaginal septum. In the present case, the pathogenesis of this localization can be explained by direct inoculation during surgery and subsequent estrogen stimulation [8]; by the metaplasia theory or may rather be an extension of the endometriosis of the vesico-uterine pouch. The metaplasia theory might apply when anterior cul-de-sac is intact [9].

The etiology and pathogenesis of endometriosis are multifactorial, but still unclear. Recent studies has demonstrated the impact of epigenetic mechanisms in the endometriosis development [10, 11]. Several factors participate in the process of differentiation, adhesion, development and persistence of ectopic endometrial cells: adhesion molecules, immune cells, pro-inflammatory cytokines, and extracellular matrix metalloproteinase [12]. It’s now proved that inflammation has an important role in the development and progression of endometriosis [10]. Vascularization is also involved in the pathogenesis of endometriosis [13].

The clinical presentation of deep endometriosis is very heterogeneous, depending on the location and extent of lesions and severity of disease, can lead to chronic pelvic pain; dysmenorrhoea; dyspareunia; infertility; bowel signs; urinary symptoms; as they may be asymptomatic. Symptoms of VVS endometriosis are often atypical, with cystalgia, urinary infections, and dysuria that characteristically flare up during menstruation [3, 14, 15]. Deep endometriosis is strongly associated with pelvic pain [16]. Painful symptoms may due to compression or infiltration of nerves by the implants. The catamenial character that is to say the exacerbation of these signs during the period is an important element of the diagnosis. In the present case, the patient had urinary symptoms without catamenial character. In the series of Kondo et al. (2011) [17], including 568 patients with deep pelvic endometriosis, the presence of a vesico-vaginal (VV) nodule was only 1.2% (7 women); however endometriosis of the uterosacral ligament accounted for 41.2% and recto-vaginal localization accounted for 18%. The average size of VV nodules in this series was 1.6 cm. In our patient’s case, the size was 6 cm (long axis). In the same series, there was excision of the nodule with vaginectomy in 2 cases. There was no complication for excision of vesicovaginal nodules. In the series of Menakaya U et al. (2016) [18]: including 192 patients, no case of involvement of the anterior vaginal or vesico-vaginal wall of deep endometriosis was diagnosed, whereas uterosacral ligament localization was the most frequently diagnosed non-intestinal form during laparoscopy (8.5% of cases). Ultrasound and pelvic MRI are the standard examinations for the assessment of deep endometriosis [19, 20]. In our patient’s case, MRI showed an aspect, suggesting a cervical leiomyoma. Positive histology will confirm the diagnosis. There must be the joint presence of epithelium and endometrial stroma or cytogenetic chorion. However, the development of ectopic endometrial tissue is highly variable depending on the response to estrogens stimulation and the age of the injury [21]. Before surgery, the clinician must evaluate the position of the mass, its anatomical relation with the ureter, bladder and bowel by imaging (preferably MRI) in case of suspicion based on a history or clinical examination. Surgical excision for an incidental finding of asymptomatic endometriosis cannot be endorsed, because it’s increased the risk of complications (injury to the bladder, ureter, bowel and blood vessels) [22]. Complete excision of deep endometriosis is effective, but is associated with a notable complication rate, especially in case of intestinal surgery [17]. Incomplete resection of lesions leads to an increased risk of recurrence and does not improve pain [17, 23]. In the present case, radical surgery was complicated by bladder breach. Nerve-sparing (NS) techniques have been integrated in surgeries for deep infiltrating endometriosis (DIE) to prevent pelvic neurological complications, due to injury of autonomic nerves. This technique decreases the risk of persistent urinary retention when compared to the conventional (non-NS) technique [24–26]. The antioxidant therapy has also proven efficacy in the treatment and mitigation of endometriosis [10].

In conclusion, Endometriosis of the anterior compartment remains relatively rare; its localization to the vesico-vaginal septum (VVS) is uncommon. With the occurrence of nonspecific cyclic urinary signs in women during periods of genital activity, endometriosis should be mentioned, especially in the presence of an antecedent of pelvic surgery.

## Data Availability

Not applicable.

## Abbreviations

VVS: Vesico vaginal septum
VV: Vesico vaginal
DIE: Deep infiltrating endometriosis
MRI: Magnetic resonance imaging
NS: Nerve-sparing
